# Association Between cMIND Diet and Depressive Symptoms Among Chinese Older Adults: A Cross-Sectional Study

**DOI:** 10.3390/nu18142349

**Published:** 2026-07-17

**Authors:** Shujuan Xiao, Shihan Zhao, Jiachi Zhang, Xingcun Zhao, Lei Shi, Xinru Li

**Affiliations:** 1 School of Health Management, Guangzhou Medical University, Guangzhou 511495, China; 2 Key Research Base for Humanities and Social Sciences of Guangdong Higher Education Institutes, Guangzhou Medical University, Guangzhou 511495, China; 3 School of Digital Economics, Nanning University, Nanning 530299, China; 4 Department of Gerontology, Simon Fraser University, Vancouver, BC V6B 5K3, Canada; 5 Gloria Gutman Gerontology Research Centre, Simon Fraser University, Vancouver, BC V6B 5K3, Canada; 6 School of Public Health, Southern Medical University, Guangzhou 510515, China; 7 School of Health Management, Southern Medical University, Guangzhou 510515, China

**Keywords:** cMIND diet, depressive symptoms, social participation, exercise, older adults

## Abstract

**Background**: With accelerated population aging, depressive symptoms in later life have become a major public health issue. The Chinese version of the MIND (cMIND) diet is a neuroprotective dietary pattern adapted to Chinese dietary habits. Previous studies have found that adherence to the cMIND diet is associated with lower levels of depressive symptoms, but the underlying statistical association patterns remain unclear. This study adopted a statistical moderated mediation framework to characterize cross-sectional associations, to examine the direct association between cMIND diet adherence and depressive symptoms, the statistical associational mediating pattern of social participation, and the exploratory moderating effect of exercise on the first segment of the statistical mediation chain. **Methods**: Data were drawn from the 2018 wave of the Chinese Longitudinal Healthy Longevity Survey (CLHLS), including 8578 adults aged 65 years and older. Cross-sectional mediation and moderated mediation analyses were conducted using Model 4 and Model 7 of the PROCESS macro, with 5000 bootstrap resamples to test the significance of cross-sectional statistical indirect effects. **Results**: cMIND diet adherence was negatively associated with depressive symptoms (*B* = −0.414, *p* < 0.01). Social participation showed a statistical partial mediation pattern in this association, with the statistical indirect effect accounting for 14.02% of the total effect. Exploratory moderated mediation analysis showed that exercise moderated the first segment of the statistical mediation chain (*B* = 0.283, *p* < 0.01). The positive association between cMIND diet adherence and social participation was stronger among exercisers (simple slope = 0.635, *p* < 0.01) than among non-exercisers (simple slope = 0.352, *p* < 0.01). Correspondingly, the indirect association between cMIND diet adherence and depressive symptoms through social participation was stronger in the exercise group (indirect effect = −0.064, 95% CI [−0.079, −0.050]) than in the non-exercise group (indirect effect = −0.036, 95% CI [−0.045, −0.026]). **Conclusions**: Among Chinese older adults, higher adherence to the cMIND diet is significantly associated with lower levels of depressive symptoms, and this association shows a partial statistical mediation pattern linked to higher social participation. The positive association between cMIND diet adherence and social participation is more pronounced among adults who engage in regular exercise, which corresponds to a more pronounced statistical indirect association with depressive symptoms through social participation. As these findings are based on cross-sectional data and reflect only associational patterns, and given the limited effect magnitude, they provide only preliminary theoretical reference for the development of intervention strategies integrating dietary improvement and social participation promotion for late-life depressive symptoms.

## 1. Introduction

With accelerating global population aging, mental health among older adults has become a major public health concern worldwide. Depressive symptoms are one of the most common mental health problems in older adults. A nationwide study showed that the prevalence of depressive symptoms among Chinese adults aged 60 years and older was as high as 43.8% [1]. Late-life depressive symptoms are often characterized by low mood, pessimism, persistent sadness, and loss of interest or pleasure in previously rewarding or enjoyable activities [2], and are significantly associated with an increased risk of suicide in older adults [3]. Therefore, identifying modifiable protective factors and exploring their potential statistical association patterns is crucial for developing effective intervention strategies.

Diet, as a highly modifiable lifestyle factor, has received increasing attention in the field of mental health in recent years. The Chinese MIND (cMIND) diet is a neuroprotective dietary pattern modified based on the traditional dietary characteristics of the Chinese population. It encourages increased intake of beneficial foods such as green leafy vegetables, berries, nuts, whole grains, soy products, and tea, while limiting the consumption of red meat, butter, and fried foods [4]. Among Chinese older adults, a cohort study based on CLHLS data from 2011 to 2018 found a significant interaction between cMIND diet adherence and the adverse effects of indoor air pollution on depressive symptoms: severe indoor air pollution exposure was associated with an increased risk of depressive symptoms in individuals with low cMIND diet adherence, while this association was significantly attenuated in those with high adherence [5]. Another study showed that cMIND diet adherence was significantly and linearly negatively correlated with both depressive and anxiety symptoms [6]. However, no significant association between the MIND diet and the risk of incident depression was observed in the Spanish SUN cohort [7]. This inconsistency in research findings suggests that the association between diet and depressive symptoms may have significant population heterogeneity, and examining only direct associations is insufficient to clarify the underlying statistical association patterns.

Social participation, a core component of the active aging framework, has been consistently linked to better mental health outcomes in older adults [8,9]. Preliminary evidence suggests that social participation may mediate the association between dietary patterns and mental health: Mediterranean dietary dimensions are positively correlated with social participation, which in turn partially mediates the association between diet and sedentary behavior [10]. From a theoretical perspective, high-quality diets may be associated with improved ability and willingness to engage in social activities, while active social interaction may also be associated with more consistent healthier dietary behaviors through peer influence and social norms [11]. Building on this evidence, we hypothesized that social participation is associated with a statistical mediation pattern in the cross-sectional association between cMIND diet adherence and depressive symptoms.

Not all older adults with high cMIND diet adherence achieve the same level of social participation, and exercise may act as a key boundary condition that strengthens the positive association between diet adherence and social participation. Our hypothesis is grounded in two interrelated theoretical perspectives. First, from a functional capacity perspective: higher dietary quality may be associated with better overall energy levels and physical fitness, but these potential benefits require sufficient physical mobility to translate into actual social engagement. Regular exercise enhances cardiorespiratory function, muscle strength, and mobility, which amplifies the extent to which dietary benefits are converted into active social participation. Empirical studies have confirmed that diet, exercise, and social participation are intercorrelated lifestyle factors that tend to cluster together [10,12], and that better physical function supports the translation of health resources into social behaviors. Second, from a behavioral synergy perspective: healthy lifestyle behaviors tend to cluster and co-occur with one another. Engagement in exercise is often accompanied by structured social scenarios such as group sports and fitness classes, creating additional social opportunities for older adults who already pay attention to dietary quality. Longitudinal evidence has shown that participation in exercise-based social groups is associated with better maintenance of healthy dietary habits [13], and that social engagement and positive emotions are shared motivators for both healthy eating and exercise [14]. This behavioral clustering pattern suggests that the association between diet adherence and social participation is more pronounced among individuals who engage in exercise, rather than the two factors operating independently. We specifically tested the moderation effect of exercise on the first segment of the statistical mediation chain, rather than alternative model structures. This model structure was selected to describe how the association between diet adherence and social participation varies by exercise status, which offers observational reference for exploring correlational patterns among multiple lifestyle factors in older adult populations.

**Figure 1:**
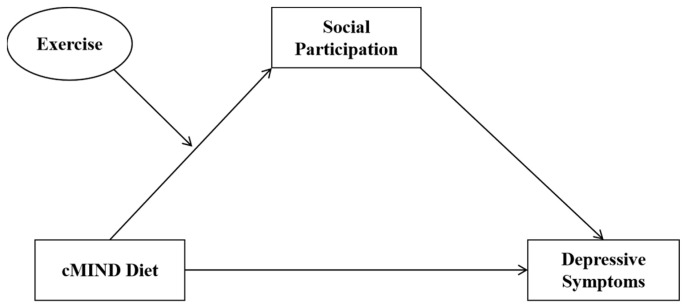
Hypothetical Model.

In summary, existing research has the following limitations: first, studies on the statistical mediation patterns underlying the association between cMIND diet adherence and depressive symptoms in older adults remain very limited, and the potential statistical mediation pattern of social participation has not been systematically verified; second, few studies have integrated cMIND diet adherence, social participation, and exercise into the same analytical framework to explore the moderating effect of physical exercise on the first segment of the statistical association chain “cMIND diet adherence → social participation → depressive symptoms”; third, relevant evidence based on nationally representative large samples of Chinese older adults is still relatively insufficient. Drawing upon a large sample data, this study adopted a statistical moderated mediation framework for cross-sectional data to systematically explore the following issues: ① the direct association between cMIND diet adherence and depressive symptoms among Chinese older adults; ② the statistical mediation pattern of social participation in this association; ③ the moderating effect of exercise on the first segment of the statistical mediation chain (cMIND diet adherence → social participation). Based on the above analysis, the following research hypotheses are proposed (see Figure 1):

**H1.**  *cMIND diet adherence is negatively associated with depressive symptoms among Chinese older adults, such that higher cMIND diet adherence is associated with lower levels of depressive symptoms*.

**H2.**  *Social participation shows a statistical partial mediation pattern in the cross-sectional association between cMIND diet adherence and depressive symptoms, such that higher cMIND diet adherence is associated with higher levels of social participation, which is in turn associated with lower levels of depressive symptoms*.

**H3.**  *Exercise moderates the positive association between cMIND diet adherence and social participation, with this association being more pronounced in older adults who engage in regular exercise*.

**H4.**  *The indirect association between cMIND diet adherence and depressive symptoms through social participation is moderated by exercise, with this statistical indirect association being more pronounced in older adults who engage in regular exercise*.

## 2. Methods

### 2.1. Data Sources

**Figure 2:**
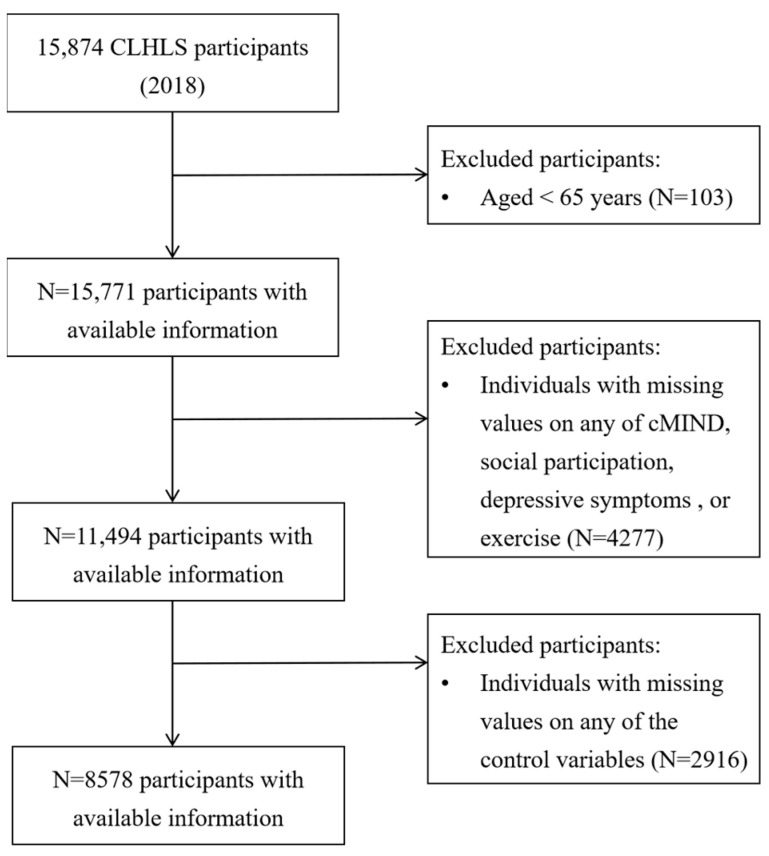
Sample selection flowchart.

Data were obtained from the Chinese Longitudinal Healthy Longevity Survey (CLHLS), conducted by the Center for Healthy Aging and Development Studies at Peking University. The survey was conducted across 23 provinces, municipalities directly under the central government, and autonomous regions nationwide using a multistage disproportionate targeted random sampling method, and collected data from older adults and their family members. The survey collected information on demographic and family characteristics, socioeconomic background and family structure, income sources and financial status, self-rated health and quality of life, cognitive function, personality and psychological characteristics, activities of daily living, lifestyle, caregiving, disease treatment, and medical expenses; for deceased participants, data on date and cause of death were collected via family member questionnaires. To date, CLHLS has completed nine waves of follow-up, accumulating detailed health data on both surviving and deceased older adults. This cross-sectional study used data from the 2018 wave. The sample selection process is illustrated in Figure 2. First, we excluded 103 participants younger than 65 years, leaving 15,771 participants with valid age information (the missing percentage for each individual variable is detailed in Supplementary Table S1). Second, we excluded 4277 participants with missing values on any of the four key variables (cMIND diet adherence, social participation, depressive symptoms, and exercise); 11,494 participants remained after this exclusion. Third, we excluded 2916 participants with missing values on any of the control variables. The final analytic sample included 8578 older adults. Comparisons of baseline characteristics between included and excluded participants are presented in Supplementary Table S2.

### 2.2. Measurements

#### 2.2.1. Dependent Variable

Depressive symptoms were assessed using the 10-item Center for Epidemiologic Studies Depression Scale (CES-D-10). The 2018 CLHLS questionnaire included two adaptations relative to the standard version: a 5-point response scale and a positively worded sleep quality item. This scale has demonstrated good applicability in Chinese older adult populations [15,16]. The recoding strategy for this adapted version was formulated with reference to two prior studies based on the CLHLS dataset, and the modified scale has been validated in comparable CLHLS analyses [17,18]. To facilitate comparability with previous studies, response options 4 and 5 were collapsed and coded as 0, option 3 as 1, option 2 as 2, and option 1 as 3; three items (“sleep quality,” “hopeful about the future,” and “as happy as when younger”) were reverse scored [19]. The total score ranges from 0 to 30. In the present study, Cronbach’s α was 0.739, KMO was 0.838, and Bartlett’s test of sphericity was significant (χ^2^ = 24,851.252, *p* < 0.001), indicating good reliability and validity.

#### 2.2.2. Independent Variable

The independent variable was cMIND diet adherence, measured using the localized cMIND diet scale developed by Huang et al. [20] based on the MIND diet index and the CLHLS food frequency questionnaire. The scale has been shown to be suitable for Chinese older adults [4,5]. To adapt to traditional Chinese dietary features and older adults’ eating habits, several key modifications were made: “olive oil” was replaced with “healthy cooking oil”; “berries” were expanded to “fresh fruit” given their low consumption frequency; red wine, which is rarely consumed by older Chinese adults, was removed; tea and garlic, which have local health evidence, were added; mushrooms or algae associated with cognitive protection were incorporated; and the type and amount of staple foods were emphasized to reflect their central role in the Chinese diet. The final scale consists of 12 items, of which three items (type of staple food, amount of staple food, and cooking oil) are scored 0 or 1, and the remaining nine items are scored 0, 0.5, or 1. The total score ranges from 0 to 12, with higher scores indicating greater cMIND diet adherence.

#### 2.2.3. Mediating Variable

The statistical mediating variable was social participation, measured by 11 activities in the CLHLS questionnaire (“Do you currently engage in/participate in the following activities?”). Response options included “almost every day,” “not every day, but at least once a week,” “not weekly, but at least once a month,” “not monthly, but occasionally,” and “never.” Following previous research [21], these were coded as 4, 3, 2, 1, and 0, respectively. Summing the scores across all activities yielded a total score ranging from 0 to 44, with higher scores indicating a higher level of social participation. In this study, Cronbach’s α was 0.701, KMO was 0.786, and Bartlett’s test of sphericity was significant (χ^2^ = 21,524.981, *p* < 0.001), demonstrating satisfactory reliability and validity.

#### 2.2.4. Moderating Variable

The statistical moderating variable was exercise, assessed by the question “Do you engage in exercise?” from the CLHLS questionnaire, and was treated as a binary variable (0 = no, 1 = yes). This binary approach follows the practice of several previous studies that have used the same CLHLS item [22,23], where exercise is operationalized as a yes/no response due to the constraints of the original questionnaire. While this single-item measure cannot capture the complexity of exercise behavior, it provides a straightforward indicator for an initial examination of exercise as a modifiable lifestyle factor. Given that exercise serves as the central moderator in the proposed cross-sectional statistical analytical framework, caution is warranted when interpreting associational findings based on this binary variable.

#### 2.2.5. Control Variables

Guided by previous studies [24,25] and the principle of selecting exogenous confounders to minimize overadjustment bias, we included variables associated with dietary quality, social participation, exercise, and depressive symptoms as controls in both the simple cross-sectional statistical mediation and moderated mediation models. The final control variables comprised age, sex, residence type, living arrangement, years of education, annual household income, marital status, smoking status, alcohol consumption, regular physical examination, and number of chronic diseases. To further verify the robustness of the findings, we additionally included self-rated health, life satisfaction, and independence in activities of daily living as covariates to conduct sensitivity analyses; the corresponding results are presented in the Supplementary Materials.

### 2.3. Analysis Strategies

First, descriptive statistics were calculated using SPSS 27.0 (IBM Corp., Armonk, NY, USA). Mann–Whitney U tests and Kruskal–Wallis H tests were used to compare the distributions of cMIND diet adherence scores and depressive symptom scores across groups defined by sex, residence type, living arrangement, marital status, smoking status, alcohol consumption, and regular physical examination. Second, Harman’s single-factor test was performed to assess common method bias. Next, normality assessments revealed that the marginal distributions of the key variables deviated from normality; therefore, Spearman correlation analysis was employed for bivariate association analysis among cMIND diet adherence, social participation, and depressive symptom scores, instead of Pearson correlation. Finally, cross-sectional statistical mediation and exploratory statistical moderated mediation analyses were conducted using the PROCESS macro (version 4.2) developed by Hayes (Model 4 and Model 7, respectively), with all control variables included in the models. All regression coefficients reported in this paper are unstandardized. It should be explicitly noted that all statistical mediation and statistical moderated mediation analyses in this study are based on cross-sectional data and represent statistical decomposition of observed cross-sectional associations; they do not provide evidence for causal mediation and cannot confirm temporal ordering among variables. A bootstrap resampling procedure with 5000 resamples was used to obtain standard errors and 95% confidence intervals for parameter estimates; if the 95% confidence interval did not contain zero, the statistical effect was considered statistically significant. We also tested for multicollinearity among all independent variables included in the regression models to verify the stability of coefficient estimates. The maximum variance inflation factor (VIF) across all models was 5.10, well below the conventional threshold of 10 [26], indicating no severe multicollinearity.

## 3. Results

### 3.1. Descriptive Statistics

A total of 8578 older adults were included in the final analysis, comprising 3955 males and 4623 females, with a median age of 82.00 years (interquartile range: 73.00, 92.00). Mann–Whitney U tests and Kruskal–Wallis H tests were conducted to compare cMIND diet adherence scores and depressive symptom scores across different demographic and health-related groups. The results are presented in Table 1. cMIND diet adherence scores differed significantly across groups defined by gender (*Z* = −13.12, *p* < 0.01), residence type (*H* = 1035.72, *p* < 0.01), living arrangement (*H* = 116.74, *p* < 0.01), marital status (*H* = 243.86, *p* < 0.01), smoking status (*Z* = −3.14, *p* < 0.01), alcohol consumption (*Z* = −8.02, *p* < 0.01), and regular physical examination (*Z* = −6.24, *p* < 0.01). Depressive symptom scores also showed significant differences across groups defined by gender (*Z* = −9.96, *p* < 0.01), residence type (*H* = 34.87, *p* < 0.01), living arrangement (*H* = 89.83, *p* < 0.01), marital status (*H* = 142.33, *p* < 0.01), smoking status (*Z* = −5.08, *p* < 0.01), alcohol consumption (*Z* = −9.12, *p* < 0.01), and regular physical examination (*Z* = −4.49, *p* < 0.01).

### 3.2. Test of Common Method Bias

Harman’s single-factor test yielded five factors with eigenvalues greater than 1, and the first factor explained 17.746% of the variance, which is below the 40% threshold [27]. This result only indicates that no severe common method bias was detected by this test, and does not confirm the complete absence of common method variance. It should be noted that Harman’s single-factor test is a preliminary diagnostic tool with well-known limitations, and is not sufficient to definitively rule out common method variance.

### 3.3. Correlation Analysis of Primary Variables

Spearman correlation analysis was used to examine cross-sectional associations among cMIND diet adherence, social participation, and depressive symptom scores. Results are presented in Table 2. cMIND diet adherence was negatively correlated with depressive symptom scores (*r* = −0.230, *p* < 0.01), indicating that higher cMIND diet adherence was associated with lower depressive symptom scores; cMIND diet adherence was positively correlated with social participation (*r* = 0.292, *p* < 0.01), indicating that higher cMIND diet adherence was associated with higher social participation; and social participation was negatively correlated with depressive symptom scores (*r* = −0.218, *p* < 0.01), indicating that higher social participation was associated with lower depressive symptom scores.

### 3.4. Test of the Mediating Effect of Social Participation

To examine whether social participation exhibits a statistical indirect association pattern in the cross-sectional association between cMIND diet adherence and depressive symptoms, cross-sectional statistical mediation analysis was performed using Model 4 of the PROCESS macro developed by Hayes for SPSS, adjusting for age, sex, residence type, living arrangement, years of education, annual household income, marital status, smoking status, alcohol consumption, regular physical examination, and number of chronic diseases. cMIND diet adherence was entered as the independent variable, social participation as the statistical mediator, and depressive symptoms as the dependent variable. A bootstrap resampling procedure with 5000 resamples was used to obtain 95% confidence intervals; statistical effects were considered statistically significant if the confidence interval did not include zero.

Results are presented in Table 3. cMIND diet adherence was negatively associated with depressive symptom scores (*B* = −0.414, *t* = −16.692, *p* < 0.01). After including social participation in the model, cMIND diet adherence remained negatively associated with depressive symptoms (*B* = −0.356, *t* = −14.272, *p* < 0.01), cMIND diet adherence was positively associated with social participation (*B* = 0.574, *t* = 16.508, *p* < 0.01), and social participation was negatively associated with depressive symptom scores (*B* = −0.101, *t* = −13.259, *p* < 0.01). Bootstrap analysis showed that social participation presented a cross-sectional statistical partial mediation pattern in the association between cMIND diet adherence and depressive symptoms, with a statistical indirect effect of −0.058 accounting for 14.02% of the total statistical effect (Table 4). Given the large sample size of this study, the statistical significance of this statistical indirect association should be interpreted with caution: the absolute value of the effect is small, reflecting a weak magnitude of association at the individual level, and the statistical significance is partly driven by the large sample.

### 3.5. Test of Moderated Mediation Model

Given the binary operationalization of exercise, this part of the analysis is exploratory in nature. To explore whether exercise moderated the above cross-sectional statistical mediation pattern, cross-sectional statistical moderated mediation analysis was conducted using Model 7 of the PROCESS macro. All models were adjusted for the aforementioned control variables. A cross-sectional statistical moderated mediation model was constructed with cMIND diet adherence as the independent variable, social participation as the statistical mediating variable, depressive symptoms as the dependent variable, and exercise as the statistical moderating variable. The statistical significance of effects was also tested using a bootstrap resampling procedure with 5000 resamples.

Results of the moderated mediation analysis are shown in Model 1 of Table 5. After adjusting for all control variables, the interaction term between cMIND diet adherence and exercise had a significant positive association with social participation level (*B* = 0.283, *t* = 4.387, *p* < 0.01), indicating that exercise significantly moderated the cross-sectional association between cMIND diet adherence and social participation level.

**Figure 3:**
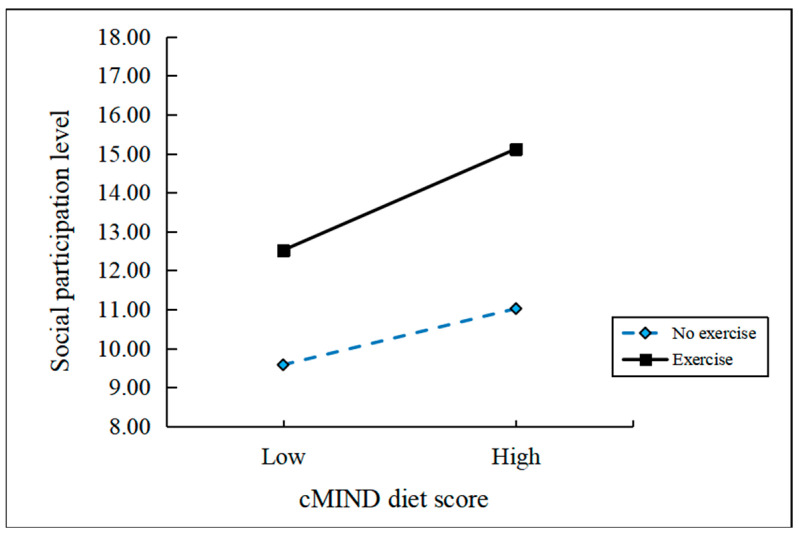
Exercise as a moderator of the relationship between cMIND diet score and social participation level.

To further clarify the pattern of the statistical moderating effect of exercise, simple slope analysis was conducted, and the results are shown in Figure 3. Among older adults with regular exercise habits, cMIND diet adherence was significantly positively associated with social participation level (Bsimple = 0.635, *t* = 11.894, *p* < 0.01, 95% CI: 0.531 to 0.740). Among older adults without regular exercise habits, the association was still significant but weaker (Bsimple = 0.352, *t* = 8.613, *p* < 0.01, 95% CI: 0.272 to 0.432).

Further conditional indirect effect analysis showed that among older adults with regular exercise habits, the cross-sectional statistical indirect association of cMIND diet adherence with depressive symptoms through social participation was significant (statistical indirect effect = −0.064, 95% CI: −0.079 to −0.050). Among older adults without regular exercise habits, the indirect association was also significant but with a smaller effect size (statistical indirect effect = −0.036, 95% CI: −0.045 to −0.026). The absolute values of the conditional statistical indirect effects in both groups were small, and the magnitude of the difference was limited. These results indicated that physical exercise significantly moderated the first segment of the statistical mediation chain. Specifically, compared with older adults without regular exercise habits, among those with regular exercise habits, higher cMIND diet adherence was cross-sectionally associated with higher levels of social participation, which in turn was associated with lower levels of depressive symptoms.

### 3.6. Sensitivity Analysis

To test the stability of the study findings, we additionally included self-rated health, life satisfaction, and independence in activities of daily living as covariates and repeated the core cross-sectional statistical mediation and moderated mediation analyses. The results of the sensitivity analysis for the statistical mediation pattern are shown in Supplementary Table S3, and the results for the statistical moderated mediation pattern are shown in Supplementary Table S4. After including the additional covariates, the core conclusions of the study remained unchanged, indicating that the findings have certain robustness.

## 4. Discussion

Drawing on a large sample of older adults from 23 provinces across China, this study adopted a cross-sectional statistical moderated mediation framework to systematically examine the association between cMIND diet adherence and depressive symptoms, and to explore the statistical association patterns involving social participation and exercise. All analyses are based on cross-sectional data and reflect only associational patterns, not causal relationships. Reverse causation is treated as a central competing explanation with equal interpretative weight throughout the paper; the statistical association chain examined represents only one theoretically plausible direction of association and is not presented as the preferred explanatory model. The main findings were as follows: (1) higher cMIND diet adherence was associated with lower depressive symptom scores; (2) higher cMIND diet adherence was associated with greater social participation, which was in turn associated with lower depressive symptom scores; and (3) as an exploratory finding, exercise strengthened the positive association between cMIND diet adherence and social participation, corresponding to a more pronounced statistical indirect association between cMIND diet adherence and depressive symptoms through social participation. These findings are discussed below.

Our study found a negative association between cMIND diet adherence and depressive symptom scores; i.e., higher dietary adherence was associated with fewer self-reported depressive symptoms. This directional association is consistent with prior longitudinal evidence on the Mediterranean diet and healthy plant-based diets [28,29]. As an observational study, our analysis cannot identify the causal mechanisms underlying this association. The theoretical biological associational accounts outlined below are hypothesized based on prior literature and were not directly measured or tested in the present study. In terms of potential theoretical biological correlates, cMIND diet adherence may be linked to lower depressive symptom levels through anti-inflammatory effects [30,31,32], modulation of the microbiota–gut–brain axis, and support of neurotrophic processes [33]. However, reverse causation provides an equally plausible account: older adults with fewer depressive symptoms are more likely to maintain a high-quality diet, as depressive symptoms are often accompanied by diminished motivation, anhedonia, and reduced self-care capacity that impairs dietary adherence. The observed association may reflect this selection process as much as—or instead of—an inverse association driven by dietary factors. These mechanistic accounts remain theoretical and speculative, and future research with biomarker data is needed to verify their role. Notably, not all plant-based dietary patterns exhibit inverse cross-sectional associations with depressive outcomes—a study also focusing on Chinese oldest-old adults found that flexitarian dietary patterns were associated with higher odds of depression and cognitive impairment [34], suggesting that overall dietary quality and nutrient adequacy may be more critical than merely emphasizing plant-based foods. Older adults with higher cMIND diet adherence, while consuming adequate plant-source foods, generally do not entirely exclude animal-source foods, which likely ensures sufficient intake of key nutrients such as vitamin B12, iron, and long-chain omega-3 fatty acids. This may partly explain the observed association with lower depressive symptom levels.

Building on this direct association, our study further found that social participation exhibited a statistical indirect association pattern in the association between cMIND diet adherence and depressive symptoms. Mediation analysis showed that older adults with higher cMIND diet adherence tended to report greater social participation, and social participation was in turn associated with lower depressive symptom scores, with a statistical indirect effect of −0.058, accounting for 14.02% of the total statistical effect. This indirect effect represents a statistical decomposition of the observed association and does not confirm a causal mediating pathway. In terms of effect magnitude, given the large sample size of this study, statistical significance alone does not equate to a strong association. The 14.02% mediation proportion indicates that social participation serves as a partial, non-dominant statistical association chain linking cMIND diet adherence to depressive symptoms. The absolute effect size is small to moderate at the individual level, with limited practical clinical relevance. While it may have potential reference value for public health practice at the population level, such population-level benefits remain hypothetical and require further verification by high-quality interventional studies. At the same time, this proportion also suggests that the majority of the association between cMIND diet and depressive symptoms operates through direct statistical associations or other unmeasured correlational factors, and social participation is only one of multiple potential dimensions of associations. This correlational pattern has been echoed in prior research. Consistent with findings from the MEDIET4ALL study on Mediterranean lifestyle patterns [10,12], dietary quality and social participation are positively correlated, and social participation acts as a modest indirect pathway linking dietary patterns to health behaviors and outcomes. Existing longitudinal evidence has also shown that sustained social participation is associated with slower increases in depressive symptoms over time [8], and that the mental health benefits of social participation vary across population subgroups [35]. The cross-sectional design of the present study cannot verify the temporal sequence of these associations, but our findings are consistent with the theoretical expectation that social participation serves as a behavioral bridge between diet and mental health. One plausible interpretation for this indirect association, which remains untested in the current study, is offered by a functional perspective. A healthy dietary pattern may be associated with better physical fitness, energy levels, and overall health perception, which may help older adults maintain and expand the breadth and frequency of their social engagement; conversely, the sense of belonging, role identity, and interpersonal support derived from active social participation represent important psychosocial resources that buffer against late-life depressive symptoms. This “dietary behavior–social participation–mental health” nexus aligns with the concept of “lifestyle behavior synergy,” which posits that multiple health behaviors such as diet, social engagement, and exercise are not isolated but rather interwoven and jointly linked to health outcomes [36].

However, an equally plausible explanation for the observed associations is reverse causation: older adults with fewer depressive symptoms are more likely to maintain healthier dietary patterns, engage in higher levels of social participation, and adhere to regular exercise. Depressive symptoms in later life are often accompanied by diminished motivation, anhedonia, and persistent fatigue, which can reduce older adults’ willingness and ability to follow a high-quality diet, seek out social interactions, and maintain consistent exercise habits. By contrast, older adults with lower depressive symptom levels generally have better behavioral initiative, more stable emotional states, and sufficient physical energy, which enable them to sustain healthy lifestyle practices including cMIND diet adherence, active social engagement, and regular activity. Given the cross-sectional observational design, the temporal sequence of these associations cannot be determined, and bidirectional associations cannot be ruled out. The statistical moderated mediation pattern outlined in this study reflects only one theoretically plausible direction; it does not confirm a unidirectional causal chain running from cMIND diet adherence to social participation and then to depressive symptoms. To mitigate such confounding, we statistically adjusted for a comprehensive set of sociodemographic, socioeconomic, behavioral, and health-related covariates in the analysis. Nevertheless, these statistical adjustments cannot fully address the reverse causation issue inherent to cross-sectional study designs, and the causal direction of the associations remains to be verified by future longitudinal research.

We further examined whether exercise moderated the first-stage association between cMIND diet adherence and social participation. Given the crude binary operationalization of exercise that cannot capture frequency, duration, intensity, type, or social context, this moderation analysis is exploratory and its interpretability is substantially limited. Results indicated that the positive association was stronger among exercisers (simple slope = 0.635) than non-exercisers (simple slope = 0.352), and the indirect association through social participation was correspondingly larger in the exercise group (indirect effect = −0.064, 95% CI: −0.079 to −0.050) than the non-exercise group (indirect effect = −0.036, 95% CI: −0.045 to −0.026). Two plausible perspectives may explain this moderation pattern, though untested by our data. From a functional capacity perspective, regular exercise is associated with better mobility and physical fitness, which may provide diet-adherent older adults with greater capacity to participate in social activities. From the psychological perspective, exercise may enhance perceived physical mastery and social comfort [37], reducing barriers to social engagement. Reverse causation again offers an equally plausible explanation: older adults with fewer depressive symptoms are more likely to sustain all three healthy behaviors—diet adherence, social participation, and regular exercise—simultaneously. This clustering of healthy behaviors among individuals with lower depressive symptoms could produce the observed moderation pattern without requiring a causal amplifying effect of exercise on the diet–social participation association. Exercise and a healthy diet may also constitute a cluster of mutually reinforcing behaviors [36], but all these interpretations remain speculative and cannot be disentangled with cross-sectional data.

This study has several strengths. It is the first to integrate cMIND diet adherence, social participation, and exercise into a single analytical framework, providing empirical evidence on the interrelationships among these factors and late-life depressive symptoms. From a purely observational standpoint, the observed co-occurrence of healthy lifestyle factors aligns with broader theoretical frameworks of lifestyle behavior clustering in later life. No actionable intervention recommendations can be drawn from the current cross-sectional findings, given the equal plausibility of reverse causation and the limited effect magnitude; the findings do not support claims of a unidirectional causal association chain and require verification in prospective and interventional research. Notably, no intervention implications are derived from the exploratory moderation analysis of exercise, as the crude binary measurement limits the interpretability and practical value of this part of the findings.

This study also has several limitations. First, the cross-sectional design precludes causal inference; as emphasized throughout the discussion, reverse causation is a central competing explanation that cannot be ruled out, and bidirectional associations are equally plausible. Future research should employ longitudinal designs to clarify temporal sequences and underlying mechanisms. Second, cMIND diet adherence, social participation, and depressive symptoms were all assessed via self-report, and common method variance may exist. Although Harman’s single-factor test did not detect severe common method bias, this test has inherent limitations and cannot fully exclude such influence; recall bias and subjective measurement error also cannot be ruled out. Third, exercise was measured only as a binary variable, without incorporating information on frequency, intensity, duration, and type, which may obscure more nuanced associations between specific exercise dimensions and the core variables. Because the CLHLS questionnaire assessed exercise with a single yes/no question, we were unable to differentiate levels or patterns of exercise. This crude measure collapses substantial heterogeneity in exercise behavior into a single dichotomous category and inevitably oversimplifies a complex health behavior. Importantly, given that exercise serves as the central moderator in our analytical model, this measurement limitation is more consequential than might appear at first glance. For this reason, we have framed the moderation analysis as exploratory rather than confirmatory. Relying on such a broad binary indicator may mask differences in how various types, intensities, or frequencies of exercise moderate the cMIND diet–social participation link, and could potentially affect the robustness of the observed interaction. Although our analysis provides a preliminary attempt—considering that the moderating pattern of exercise in the cMIND diet–depressive symptoms association chain has rarely been explored—future research should prioritize multidimensional assessments of exercise (e.g., frequency, intensity, duration, type) to examine whether these dimensions differentially moderate the statistical mediation pattern and to verify the stability of the current findings. Fourth, residual confounding remains a notable limitation despite the expanded set of adjusted covariates. Although we have included a comprehensive range of sociodemographic, socioeconomic, behavioral, and health-related confounders, unmeasured factors may still exist and contribute to residual confounding, which could influence the observed associations. Meanwhile, our covariate selection strategy was designed to mitigate overadjustment bias by excluding potential intermediate variables in the theoretical associational chain; while this approach aligns with standard epidemiological practice, it cannot fully eliminate concerns related to covariate specification. To address this concern, we conducted sensitivity analyses by additionally including self-rated health, life satisfaction, and independence in activities of daily living as covariates, and the core findings remained stable. Nevertheless, residual confounding cannot be completely ruled out. Fifth, the complete-case analysis carries a non-negligible risk of selection bias. A large proportion of participants were excluded due to missing values on key variables and control variables, and baseline characteristic comparisons confirmed that missingness was not completely random. This non-random missing pattern may distort sample composition and reduce sample representativeness, and the findings should be interpreted with caution when generalized to the broader older adult population. Owing to the constraints of the PROCESS macro-based core analytical framework, sensitivity analyses such as multiple imputation and inverse probability weighting were not conducted in this study, and the robustness of the findings against missing data bias cannot be fully verified. Future studies may adopt multiple imputation or other robust missing data handling methods to mitigate this bias and enhance the robustness of results. Sixth, cognitive function was not included as a covariate in this study. The Mini-Mental State Examination (MMSE) data in the CLHLS dataset were not used due to copyright-related restrictions, and there are no alternative cognitive status indicators available in the dataset. Since cognitive function may simultaneously influence diet quality, social participation, exercise, and depressive symptoms, the omission of this variable may lead to residual confounding and is a limitation of the study. Last, besides exercise, other potential moderating variables (e.g., educational attainment, socioeconomic status) were not included in the analysis, and subsequent studies could explore these to achieve a more comprehensive understanding of the complex relationships among these factors.

## 5. Conclusions

This cross-sectional study found that, among Chinese older adults, cMIND diet adherence was negatively associated with depressive symptom scores, and social participation presented a cross-sectional statistical partial mediation pattern in this association. As an exploratory finding limited by the binary operationalization of exercise, exercise moderated the first half of this statistical mediation chain: compared with older adults who did not engage in regular exercise, older adults who exercised regularly showed a stronger positive association between cMIND diet adherence and social participation, and a more pronounced statistical indirect association between cMIND diet adherence and depressive symptoms through social participation. Notably, the overall effect sizes of the direct and statistical indirect associations are limited, and their practical clinical relevance remains to be clarified. These patterns of association collectively suggest that higher dietary quality, active social participation, and regular exercise exhibit a clustered cross-sectional association with lower levels of late-life depressive symptoms. Given the observational cross-sectional design, causal inferences cannot be drawn, and future longitudinal and interventional studies are warranted to verify the temporal sequence and potential mechanisms among these variables.

## Figures and Tables

**Table 1:** Descriptive Statistics and Group Differences.

Variables	Variable Assignment	M (P_25_, P_75_)/ N (Percentage)	cMIND Diet		Depressive Symptoms	
			*Z*	*H*	*Z*	*H*
Age	Continuous type	82.00 (73.00, 92.00)	—		—	
Gender	0 = Female	4623 (53.89%)	−13.12 **	—	−9.96 **	—
	1 = Male	3955 (46.11%)				
Residence	0 = rural	3525 (41.09%)	—	1035.72 **	—	34.87 **
	1 = city	2295 (26.76%)				
	2 = town	2758 (32.15%)				
Living Arrangement	0 = in an institution	290 (3.38%)	—	116.74 **	—	89.83 **
	1 = with household member(s)	6927 (80.75%)				
	2 = alone	1361 (15.87%)				
Years of Education	Continuous type	3.00 (0.00, 6.00)	—		—	
Annual Household Income	(10,000 CNY) Continuous type	3.00 (0.90, 8.00)	—		—	
Marital Status	0 = Married	4118 (48.01%)	—	243.86 **	—	142.33 **
	1 = Divorced	28 (0.33%)				
	2 = Widowed	4368 (50.92%)				
	3 = Unmarried	64 (0.75%)				
Smoking Status	0 = no	7210 (84.05%)	−3.14 **	—	−5.08 **	—
	1 = yes	1368 (15.95%)				
Alcohol Consumption	0 = no	7255 (84.58%)	−8.02 **	—	−9.12 **	—
	1 = yes	1323 (15.42%)				
Physical Examination	0 = no	2463 (28.71%)	−6.24 **	—	−4.49 **	—
	1 = yes	6115 (71.29%)				
Number of Chronic Diseases	Continuous type	1.00 (0.00, 2.00)	—		—	

Note: ** *p* < 0.01; *Z*: represents the Mann–Whitney U test; *H*: represents the Kruskal–Wallis H test.

**Table 2:** Correlation analysis of key variables.

	M ± SD	cMIND Diet	Depressive Symptoms	Social Participation
cMIND Diet	5.58 ± 2.05	1.000		
Depressive Symptoms	7.28 ± 4.46	−0.230 **	1.000	
Social Participation	11.62 ± 7.41	0.292 **	−0.218 **	1.000

** Significant at *p* < 0.01.

**Table 3:** Results of the mediation analysis.

Criterion	Predictors	*R* ^2^	*B*	SE	*t*	95% CI
Depressive Symptoms	cMIND Diet	0.097	−0.414	0.025	−16.692 **	[−0.462, −0.365]
Social Participation	cMIND Diet	0.356	0.574	0.035	16.508 **	[0.506, 0.643]
Depressive Symptoms	cMIND Diet	0.116	−0.356	0.025	−14.272 **	[−0.404, −0.307]
	Social Participation	0.116	−0.101	0.008	−13.259 **	[−0.116, −0.086]

** *p* < 0.01.

**Table 4:** Summary of Mediation Effects.

Effect Type	Effect	LLCI	ULCI	Percentage of Total Effect
Total Effect	−0.4136	−0.462	−0.365	100%
Direct Effect	−0.3556	−0.404	−0.307	85.98%
Indirect Effect	−0.0580	−0.070	−0.047	14.02%

**Table 5:** Results of moderated mediation analysis.

Variables	Model 1: Social Participation			Model 2: Depressive Symptoms		
	*B*	*t*	95%CI	*B*	*t*	95%CI
cMIND Diet	0.352	8.613 **	[0.272, 0.432]	−0.356	−14.272 **	[−0.404, −0.307]
Social Participation				−0.101	−13.259 **	[−0.116, −0.086]
Exercise	1.937	4.844 **	[1.153, 2.722]			
cMIND Diet × Exercise	0.283	4.387 **	[0.157, 0.410]			
*R* ^2^	0.406			0.116		
*F*	417.638 **			86.007 **		

** *p* < 0.01.

## Data Availability

The datasets analyzed in this study are not publicly available due to ongoing related research but are available from the corresponding author upon reasonable request. The original CLHLS datasets are accessible to qualified researchers via the Peking University Open Research Data Platform after formal application and approval.

## Supplementary Materials

The following supporting information can be downloaded at: https://www.mdpi.com/article/10.3390/nu18142349/s1, Table S1: Pattern of missing values. Table S2: Baseline Characteristics of Retainers and Attritors. Table S3: Results of the mediation analysis. Table S4: Results of moderated mediation analysis.
