# Supplementary material for: Association Between cMIND Diet and Depressive Symptoms Among Chinese Older Adults: A Cross-Sectional Study
Source: Nutrients. 2026 Jul 17;18(14):2349. doi: 10.3390/nu18142349 (PMC13414518; doi:10.3390/nu18142349)
Supplement: Supplementary file 1 [file nutrients-18-02349-s001.zip › nutrients-4396940-supplementary.pdf]

Table S1. Pattern of missing values.

Variables	Pattern of missing values (n / %)
Age	15771 (0.00%)
Gender	15771 (0.00%)
Residence	15771 (0.00%)
Living Arrangement	314 (2.03%)
Years of Education	2334 (17.37%)
Annual Household Income	1510 (10.59%)
Marital Status	254 (1.64%)
Smoking Status	253 (1.63%)
Alcohol Consumption	348 (2.26%)
Physical Examination	179 (1.15%)
Number of Chronic Diseases	614 (4.05%)
cMIND Diet	841 (5.63%)
Depressive Symptoms	3398 (27.46%)
Social Participation	449 (2.93%)
Exercise	338 (2.19%)

Table S2. Baseline Characteristics of Retainers and Attritors.

Characteristics	Sample Groups (n / %)		Z / χ^2
	Attritors (n=7193)	Retainers (n=8578)	
Age (continuous variable)[a]	—	—	-30.529**
Gender			49.811**
0=Female	4279 (59.49%)	4623 (53.89%)	
1=Male	2914 (40.51%)	3955 (46.11%)	
Living Arrangement[b]			5.935
0=in an institution	282 (3.92%)	290 (3.38%)	
1=with household member(s)	5488 (76.30%)	6927 (80.75%)	
2=alone	1109 (15.42%)	1361 (15.87%)	
Marital Status[b]			408.816**
0=Married	2219 (30.85%)	4118 (48.01%)	
1=Divorced	23 (0.32%)	28 (0.33%)	
2=Widowed	4625 (64.30%)	4368 (50.92%)	
3=Unmarried	72 (1.00%)	64 (0.75%)	

Note: [a] denotes the Mann-Whitney U test; [b] denotes missing values; **Significant at P <0.010; *Significant at P <0.050.

Table S3. Results of the mediation analysis.

Criterion	Predictors	R^2	*B*	SE	*t*	95% CI
Depressive Symptoms	cMIND Diet	0.293	-0.203	0.022	-9.145**	[-0.246, -0.159]
Social Participation	cMIND Diet	0.393	0.517	0.034	15.085**	[0.449, 0.584]
Depressive Symptoms	cMIND Diet	0.299	-0.171	0.022	-7.666**	[-0.215, -0.127]
	Social Participation	0.299	-0.060	0.007	-8.768**	[-0.074, -0.047]

**P<0.01

Table S4. Results of moderated mediation analysis.

Variables	Model 1：Social Participation			Model 2：Depressive Symptoms		
	B	*t*	95%CI	*B*	*t*	95%CI
cMIND Diet	0.331	8.167**	[0.251, 0.410]	-0.170	-7.587**	[-0.214, -0.126]
Social Participation				-0.060	-8.703**	[-0.074, -0.047]
Exercise	1.647	4.212**	[0.881, 2.414]			
cMIND Diet × Exercise	0.271	4.301**	[0.147, 0.394]			
R^2	0.432			0.301		
F	384.036**			230.995**		

**P<0.01
